# Rooting phylogenetic trees under the coalescent model using site pattern probabilities

**DOI:** 10.1186/s12862-017-1108-7

**Published:** 2017-12-19

**Authors:** Yuan Tian, Laura Kubatko

**Affiliations:** 1 Department of Evolution, Ecology, and Organismal Biology, The Ohio State University, 318 W. 12th Avenue, Columbus, 43210 OH USA; 2 Department of Statistics, The Ohio State University, 404 Cockins Hall, 1958 Neil Avenue, Columbus, 43210 OH USA

**Keywords:** Phylogeny, Root, Site pattern probability, Outgroup, Coalescent

## Abstract

**Background:**

Phylogenetic tree inference is a fundamental tool to estimate ancestor-descendant relationships among different species. In phylogenetic studies, identification of the root - the most recent common ancestor of all sampled organisms - is essential for complete understanding of the evolutionary relationships. Rooted trees benefit most downstream application of phylogenies such as species classification or study of adaptation. Often, trees can be rooted by using outgroups, which are species that are known to be more distantly related to the sampled organisms than any other species in the phylogeny. However, outgroups are not always available in evolutionary research.

**Methods:**

In this study, we develop a new method for rooting species tree under the coalescent model, by developing a series of hypothesis tests for rooting quartet phylogenies using site pattern probabilities. The power of this method is examined by simulation studies and by application to an empirical North American rattlesnake data set.

**Results:**

The method shows high accuracy across the simulation conditions considered, and performs well for the rattlesnake data. Thus, it provides a computationally efficient way to accurately root species-level phylogenies that incorporates the coalescent process. The method is robust to variation in substitution model, but is sensitive to the assumption of a molecular clock.

**Conclusions:**

Our study establishes a computationally practical method for rooting species trees that is more efficient than traditional methods. The method will benefit numerous evolutionary studies that require rooting a phylogenetic tree without having to specify outgroups.

## Background

Phylogenetic tree inference is a fundamental framework in which to estimate the ancestor-descendant relationships among different species. Currently, the amount of DNA sequence data is increasing dramatically, and more accurate and efficient methods are required to estimate phylogenetic trees using these data. Evolutionary relationships can be analyzed at two distinct levels (gene trees and species trees), and it is not necessary for the gene trees and species trees to agree with one another [1–6]. Incomplete lineage sorting (ILS) is considered to be one of the major factors that causes disagreement between species trees and gene trees, and thus ILS has a critical effect on estimation of the species tree using large multi-locus data sets [1, 7–13].

In many species tree inference approaches, gene trees are estimated first and are assumed as known in the following analysis [14–22]. However, such gene trees are often not fully informative, because they may be based on short sequences with few variable sites [23]. As a result, the gene tree estimation errors may potentially become a severe issue in species tree inference. Some coalescent inference methods, such as ASTRAL, do not directly infer the root of the estimated species phylogeny [14, 15]. Still other coalescent inference methods (MP-EST, NJst) require rooted gene trees as the input in order to estimate a rooted species tree [18, 22]. However, ancestor (rooting) identification is essential for complete understanding of the evolutionary relationships. Rooted trees benefit most downstream applications of phylogenies, such as species classification and comparative biology. In many cases, trees can be rooted using outgroups, which are known species that are more distantly related to the sampled organisms than any other species in the phylogeny. However, outgroups are not always available in evolutionary research. For instance, in numerous unresolved evolutionary questions such as animal evolution [24, 25], placental mammal evolution [26–29], prokaryotic evolution [30, 31], and even the beginnings of life [31, 32], it is difficult to specify appropriate outgroups, because of issues such as long branch attraction [33] and variation in the substitution process [34]. Thus, rooting methods in the absence of outgroups are often necessary for phylogenetic inference. While other methods for rooting trees have been proposed (i.e., midpoint rooting, rooting with a molecular clock, as well as Bayesian versions of these [35]), each has its own drawbacks [36] and none were designed for use on species-level phylogenies that are subject to incomplete lineage sorting. For a recent review of rooting methods, see [37].

In our study, we develop a new method for rooting species tree under the coalescent model, by developing a series of hypothesis tests for rooting quartet phylogenies using site pattern probabilities. More specifically, the site pattern probabilities of every four-taxon quartet are used to construct rooted species trees based on an unrooted species tree topology. Our study establishes a computationally practical method of rooting species trees in the absence of an outgroup. Since a rooted species tree will provide more information about evolutionary relationships, the new method will benefit numerous evolutionary studies that require rooting a phylogenetic tree without having to specify outgroups.

## Methods

The coalescent process [1, 10, 38] is a retrospective model of population genetics that is commonly used to model incomplete lineage sorting (ILS). Based on tracing the evolutionary history of sampled genes by considering the time from the present back to their most recent common ancestor [39], the coalescent model is used as the basis for different methods to estimate species trees (e.g. [18, 40–43]; reviewed in Edwards [44]). Under the coalescent model, our method uses relationships among the expected site pattern probabilities to develop a method to root phylogenetic trees. We define a *coalescent independent site* as a column in a DNA alignment for which all nucleotides have evolved from a common ancestor according to some evolutionary process. Coalescent independent sites are assumed to freely recombine with one another.

### Method for rooting phylogenetic trees by site pattern probabilities

In a four-taxon species tree, there are 4^4^=256 possible site patterns. Let $p_{i_{A}i_{B}i_{C}i_{D}}, (i_{a} \in \{A, C, G, T\}, a = A, B, C, D)$ represent the probabilities of each site pattern *i*_*A*_ *i*_*B*_ *i*_*C*_ *i*_*D*_, where *i*_*a*_ refers to the nucleotide at tip *a* of the four-taxon species tree. Any site pattern probability of a rooted four-taxon species tree under the molecular clock assumption can be classified into one of 15 categories:

*p*_*xxxx*_, *p*_*xxxy*_, *p*_*xxyx*_, *p*_*xyxx*_, *p*_*yxxx*_, *p*_*xyxy*_, *p*_*xyyx*_, *p*_*xxyy*_, *p*_*xyxz*_, *p*_*xyzx*_, *p*_*yxxz*_, *p*_*yxzx*_, *p*_*xxyz*_, *p*_*yzxx*_, *p*_*xyzw*_ where w, x, y, and z denote different nucleotide states. To explore the rooting position for an unrooted four-taxon tree, which can then be used to infer the root position on a larger phylogenetic tree, we develop a series of hypothesis tests, based on expected site pattern probabilities (Table 1).

**Table 1:** Relationships expected among site pattern probabilities for various root positions. See Fig. 1 for depiction of root positions ① - ⑤

Rooting position	Expected relationships
①	*p*_*yxxx*_>*p*_*xyxx*_, *p*_*xxxy*_=*p*_*xxyx*_
②	*p*_*xyxx*_>*p*_*yxxx*_, *p*_*xxxy*_=*p*_*xxyx*_
③	*p*_*xxyx*_>*p*_*xxxy*_, *p*_*xyxx*_=*p*_*yxxx*_
④	*p*_*xxxy*_>*p*_*xxyx*_, *p*_*xyxx*_=*p*_*yxxx*_
⑤	*p*_*xyxx*_=*p*_*yxxx*_, *p*_*xxxy*_=*p*_*xxyx*_

**Fig. 1:**
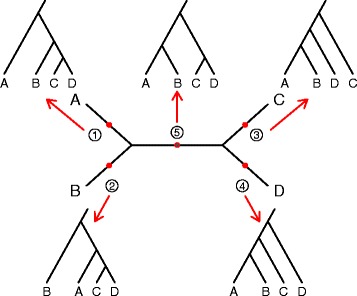
Example species trees. All possible splits of a four-leaf unrooted species tree are shown in the center of the figure. Five possible root positions are labelled on the unrooted tree, with arrows pointing to the five possible rooted species trees with four leaves

These hypothesis tests are derived from the equivalence of site pattern probabilities in a four-taxon phylogenetic tree. For instance, if the rooting position is ① (Fig. 1), it is clear that species C and D have equal probabilities of mutating under the molecular clock assumption. Therefore, *p*_*xxxy*_=*p*_*xxyx*_. On the other hand, species A can be considered as an outgroup in these four species, and the site pattern *yxxx* is more likely than *xyxx*, thus it is easy to see that *p*_*yxxx*_>*p*_*xyxx*_. Similarly, we can write expected relationships for the other four root positions (Table 1, Fig. 1). Note that *p*_*xyxz*_=*p*_*xyzx*_, and *p*_*yxxz*_=*p*_*yxzx*_, could also be used, but our preliminary results suggested that the values of *p*_*xyxx*_, *p*_*yxxx*_, *p*_*xxxy*_ and *p*_*xxyx*_ are larger than *p*_*xyxz*_, *p*_*xyzx*_, *p*_*yxxz*_, and *p*_*yxzx*_, thereby giving better performance when estimated from empirical data.

Note that the analytical derivation of the site pattern probabilities arising from the coalescent model under the JC69 model is given by Chifman and Kubatko [45]. It is not surprising that under the JC69 model, many site pattern probabilities are identical due to the assumption of equal base frequencies and identical nucleotide substitution rates. Indeed, site pattern probabilities within each category described above are identical under the JC69 model. Therefore, based on the precise formulas for the site pattern probabilities derived by Chifman and Kubatko [45], the relationships in Table 1 can be mathematically proved under the JC69 model. Analytical proof is not given for other nucleotide substitution models due to increasing complexity in computing caused by unequal base frequencies and varying nucleotide substitution rates. However, with the clock assumption, it is still reasonable to apply the method under other nucleotide substitution models, because the probabilities of having specific classes of mutations (for example, a change from *A* to *C*) are identical for sister species and are always proportional to branch length under any nucleotide substitution model. The performance of our rooting method under varying nucleotide substitution models will be tested using simulation studies.

### Formal hypothesis tests

To determine the root position, we first set up two distinct hypothesis tests:

$$\text{ Test 1:}\; H_{0}: p_{yxxx}=p_{xyxx}\ \text{vs.}\ H_{1}: p_{yxxx} \neq p_{xyxx}, $$

and

$$\text{ Test 2:}\; H_{0}: p_{xxyx}=p_{xxxy}\ \text{vs.}\ H_{1}: p_{xxyx} \neq p_{xxxy}. $$

Note that there are 12 possible site pattern probabilities within each category of *yxxx*, *xyxx*, *xxyx*, or *xxxy*. For example, the site patterns *ACCC*, *GCCC*, and *AGGG* (and 9 others) all have the form *yxxx*. Thus, rather than consider all 256 of the possible site patterns, we consider five categories of site patterns: *yxxx*, *xyxx*, *xxyx*, *xxxy*, and “other”, where the category “other” refers to the remaining 208 site patterns that don’t satisfy one of the first four forms. Let ***X***=[*X*_1_,*X*_2_,*X*_3_,*X*_4_,*X*_5_] denote the vector of the counts for each of these five categories, and ***q***=[*q*_1_,*q*_2_,*q*_3_,*q*_4_,*q*_5_] denote the vector of category probabilities. Then ***X***∼ Multinomial(*M*, ***q***), where *M* is the number of coalescent independent sites. Under the assumption of a multinomial distribution, we can compute the mean and variance of each count and the covariance between them. We note that

$$\begin{array}{@{}rcl@{}} E(X_{s}) =& Mq_{s} & s = 1, 2, 3, 4, 5,\\ Var(X_{s}) =& {Mq}_{s}(1-q_{s}) & s = 1, 2, 3, 4, 5,\\ cov(X_{s},X_{t}) =& -{Mq}_{s}q_{t} & s = 1, 2, 3, 4, 5; t = 1, 2, 3, 4, 5; s \neq t.\\ \end{array} $$

Note that the *q*_*i*_ are defined above to be the probability of observing a site pattern from category *i*, *i*=1,2,3,4,5. We estimate this probability by the frequency observed in the data. To be specific, we have the following

1 $$\begin{array}{*{20}l} q_{1}&=\sum\limits_{\substack{i,j \in \{A,C,G,T\}\\ i \neq j}} p_{jiii}, & \hat{q}_{1}&=\frac{1}{M}\sum\limits_{\substack{i,j \in \{A,C,G,T\}\\ i \neq j}} N_{jiii},  \\ q_{2}&=\sum\limits_{\substack{i,j \in \{A,C,G,T\}\\ i \neq j}} p_{ijii}, & \hat{q}_{2}&=\frac{1}{M}\sum\limits_{\substack{i,j \in \{A,C,G,T\}\\ i \neq j}} N_{ijii}, \\ q_{3}&=\sum\limits_{\substack{i,j \in \{A,C,G,T\}\\ i \neq j}} p_{iiji}, & \hat{q}_{3}&=\frac{1}{M}\sum\limits_{\substack{i,j \in \{A,C,G,T\}\\ i \neq j}} N_{iiji},  \\ q_{4}&=\sum\limits_{\substack{i,j \in \{A,C,G,T\}\\ i \neq j}} p_{iiij}, & \hat{q}_{4}&=\frac{1}{M}\sum\limits_{\substack{i,j \in \{A,C,G,T\}\\ i \neq j}} N_{iiij},  \end{array} $$

where *N*_*jiii*_ denotes the number of times site pattern *jiii* occurs in the observed data, for example.

Thus, we have:

2 $$\begin{array}{*{20}l} {}E(\hat{q}_{1}-\hat{q}_{2})&= q_{1}-q_{2} \end{array} $$

3 $$\begin{array}{*{20}l} {}Var(\hat{q}_{1}-\hat{q}_{2})&=\frac{1}{M}\left[q_{1}(1-q_{1})+q_{2}(1-q_{2})+2q_{1}q_{2}\right] \end{array} $$

4 $$\begin{array}{*{20}l} {}E(\hat{q}_{3}-\hat{q}_{4})&= q_{3}-q_{4} \end{array} $$

5 $$\begin{array}{*{20}l} {}Var(\hat{q}_{3}-\hat{q}_{4})&=\frac{1}{M}\left[q_{3}(1-q_{3})+q_{4}(1-q_{4})+2q_{3}q_{4}\right] \end{array} $$

Now, using Eqs. (2) - (5), substituting the estimated site pattern probabilities into Eqs. (3) and (5), we can compute test statistics for both hypothesis tests:

6 $$ Z_{1}=\frac{\hat{q}_{1}-\hat{q}_{2}} {\sqrt{\frac{1}{M}\left[\hat{q}_{1}(1-\hat{q}_{1})+\hat{q}_{2}(1-\hat{q}_{2})+2\hat{q}_{1}\hat{q}_{2}\right]}},  $$

7 $$ Z_{2}=\frac{\hat{q}_{3}-\hat{q}_{4}} {\sqrt{\frac{1}{M}\left[\hat{q}_{3}(1-\hat{q}_{3})+\hat{q}_{4}(1-\hat{q}_{4})+2\hat{q}_{3}\hat{q}_{4}\right]}},  $$

Under the null hypothesis in Test 1 that *p*_*yxxx*_=*p*_*xyxx*_, *Z*_1_∼*N*(0,1) when *M* is large. Similarly, *Z*_2_∼*N*(0,1) under the null hypothesis in Test 2 that *p*_*xxyx*_=*p*_*xxxy*_ when *M* is large. Therefore, our rooting method can be applied by checking the test results and values of *Z*_1_ and *Z*_2_. More specifically, for example, if we reject Test 1, accept Test 2, and *Z*_1_>0, we can conclude that the root position is ①. Similarly, the other test results and their conclusions are summarized in Table 2. Note that significance levels for the two tests, *α*_1_ and *α*_2_, must be selected. In our study, we choose the significance levels *α*_1_=*α*_2_=0.025. The significance levels can be adjusted for different studies. The performance of the rooting method are evaluated by simulation studies, as described below.

**Table 2:** Test results and conclusions for rooting a four-taxon phylogenetic tree

Test 1	Test 2	*Z*_1_	*Z*_2_	Inferred rooting position
Reject	Accept	>0	NA	①
Reject	Accept	<0	NA	②
Accept	Reject	NA	>0	③
Accept	Reject	NA	<0	④
Accept	Accept	NA	NA	⑤
Reject	Reject	NA	NA	No conclusion

### Simulation studies

Three sets of simulation studies were used to examine the performance of our method to root the species quartets. All simulation studies include DNA sequence data simulated from four-taxon species trees. More specifically, different numbers of gene trees are generated from the species trees with COAL [13], then coalescent independent sites or multi-locus DNA sequences are simulated by using Seq-Gen [46]. The simulation process is repeated 500 times to generate 500 independent data sets, the rooting method is applied to each data set, and the power (proportion of the 500 data sets for which the correct conclusion is made) for each simulation setting is recorded.

The first set of simulation studies is designed to assess the performance of our method for coalescent independent sites when the molecular clock holds. Two groups of species trees with “long” and “short” branch lengths are used to simulate the data. Each group contains two species trees that have the same unrooted topology, but different rooting positions. Note that though there are five rooting positions for a 4-taxon species tree, four of them lead to asymmetric rooted trees (① - ④ in Fig. 1), and the rooting method is identical for them. Thus, only ① and ⑤ are used in our simulation studies. For the “long branch lengths” group, the two species trees used are (*A*:3.0,(*B*:2.0,(*C*:1.0,*D*:1.0):1.0)) and ((*A*:0.8,*B*:0.8):2.2,(*C*:1.2,*D*:1.2):1.8). The two species trees in the “short branch lengths” group have the same topologies as in the “long branch lengths” group, but all branch lengths are scaled by 0.5 (all branch lengths in our study are measured in coalescent units). A varying number of gene trees (5000, 10,000, 20,000, 100,000) are simulated from each species tree. To convert between coalescent units and mutation units, a value of *θ*=4*N*_*e*_ *μ*=0.05 is used to scale the branch lengths of the simulated gene trees. The gene trees are then used to simulate coalescent independent sites (one site for each gene tree) with the program Seq-Gen [46] under the JC69, HKY85 (Seq-Gen command: -mHKY -t 3.0 -f 0.3 0.2 0.2 0.3), and GTR+I+ *Γ* (Seq-Gen command: -mGTR -r 1.0 0.2 10.0 0.75 3.2 1.6 -f 0.15 0.35 0.15 0.35 -i 0.2 -a 5.0 -g 3) models. For each parameter setting, 500 replications are simulated to estimate the root position, and the proportion for which the correct conclusion is reached is recorded as the power of the study.

The second set of simulation studies focuses on multi-locus DNA sequence data instead of coalescent independent sites. In the first set of simulation studies, we simulate a number of gene trees, and only one site is simulated under each gene tree as a coalescent independent site. However, we also wish to explore the performance of our method for multi-locus data. The simulation studies have similar parameter settings to the first set of simulations, but instead of a single site, a DNA sequence of 500 base pairs is simulated from each gene tree using Seq-Gen [46]. The number of gene trees is adjusted to (50,100,200,1000) to keep the total number of sites identical to that used in the first set of simulations.

The third set of simulation studies is designed to assess the robustness of the procedure when the assumptions are violated. First, we consider the case in which the molecular clock assumption is violated for coalescent independent sites and the “long” species tree setting. We wrote custom python scripts to simulate gene trees from both the symmetric and asymmetric species trees for which the branch leading to taxon *A* is extended, and for which the branch leading to taxon *C* is extended in the asymmetric case. We consider varying the length of the branches leading to either taxon *A* or taxon *C* from their original values of 1.0 in the first set of simulation studies to the values 1.1, 1.2, 1.3, 1.4, or 1.5. After simulating gene trees from these non-clock species trees, the procedure was identical to that above. Specifically, we simulate sequence data under the coalescent independent sites and JC69 models using Seq-Gen [46] and record how many times the correct tree was inferred. Second, we consider the case in which the true tree is a star phylogeny (i.e., there is no root to be identified), and record whether the method prefers a particular root in this case. Intuitively, we might expect the method to prefer the symmetric rooting along branch ⑤ since the two null hypotheses specified by Tests 1 and 2 in “Formal hypothesis tests” section will be satisfied for the star phylogeny with the symmetric rooting position when the molecular clock holds.

### Application to larger species trees

**Fig. 2:**
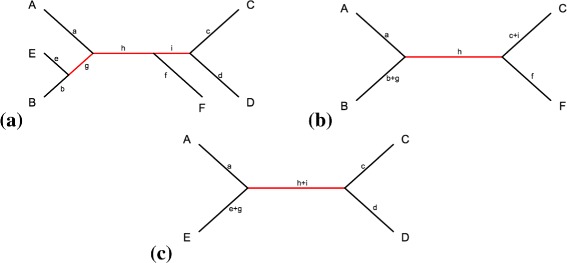
Example of scoring potential root psitions on larger phylogenies. **a** A six-leaf unrooted species tree. The branches are coded from *a* to *i*, and the internal branches are highlighted in red; **b** An example species quartet ABCF. If the root is determined to be on branch *a*, *f* or *h*, the corresponding branch on the tree in **a** will get score 1. If the root is determined to be on branch *b*+*g* (or *c*+*l*), branches *b* and *g*, (or *c* and *l*) in **a** will each get score 0.5. **c** An example species quartet AECD. If the root is determined to be on branch *a*, *c* or *d*, the corresponding branch in **a** will get score 1. Otherwise, if the root is determined to be on branch *e*+*g* (or *h*+*l*), branches *e* and *g* (or *h* and *l*) will each get score 0.5

To examine the performance of our rooting method for larger taxon samples, we assume that the unrooted tree has been previously estimated. In our example, we estimate the species tree using SVDQuartets, a full-data coalescent-based method based on site pattern probabilities, and we label each branch with a particular code (Fig. 2 a). Our method works by randomly selecting a subset of four species from the *n* species under study, and determining the root position, as shown in Fig. 1. This is repeated many times, for many randomly selected quartets. If the number of taxa is not too large, all quartets can be considered; otherwise, a random sample can be taken. Note that there are multiple correlated hypothesis tests for a species tree with more than 4 taxa. To handle the issue of multiple tests, we use the Bonferroni correction. When an overall *α*-level test for an *n*-taxon species tree is desired, we use $\alpha / {n \choose 4}$ as the critical value in the tests, when all quartets are sampled.

To determine the root of a given species tree with more than 4 taxa after the selected quartets have been evaluated, we develop a method to combine the results from the individual quartet tests. This method assigns a weighted score for each branch based on the results of the analysis of the individual quartets. Suppose a particular species quartet is composed of five branches (Fig. 2 a, b), where any branch contains one or more coded branches as shown in Fig. 2 a. Denote the number of the coded branches within the five branches as *n*_1_, *n*_2_, *n*_3_, *n*_4_, and *n*_5_, respectively. Once a branch *n*_*i*_(*i*=1,2,3,4,5) is determined to contain the root, any coded branch within the determined branch has score $\frac {1}{n_{i}}$, while other branches have score 0. Two examples are shown in Fig. 2 b and c. The branch with the highest summed scores over all quartets evaluated will be selected as the location of the root.

## Results

### Accuracy of the method for rooting phylogenetic trees

**Fig. 3:**
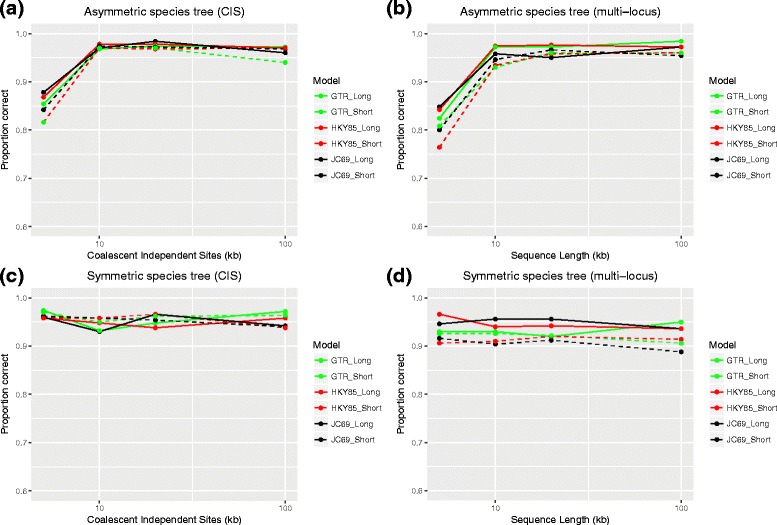
Accuracy of the rooting method when the molecular clock assumption holds. In each panel, the *x*-axis denotes the data size (kb), and the *y*-axis shows the proportion of the data sets for which the correct rooting position is selected in a total of 500 simulations. From left to right, each column represents one set of simulation studies as described in the Methods section. **a**, **c**: The first set of simulation studies (coalescent independent sites); **b**, **d**: The second set of simulation studies (multi-locus DNA). From top to bottom, each row represents one possible split of a four-leaf unrooted species tree denoted by ① and ⑤ as in Fig. 1. **a**, **b**: Root position at ①, **c**, **d**: Root position at ⑤. Solid lines in each panel represents the species trees in the “Long branch lengths” group, while the species trees in the “Short branch lengths” group are denoted by dashed lines. In the simulation studies, DNA sequences data is simulated under the JC69 (black), HKY (red), and GTR+I+ *Γ* (green) models, respectively

**Fig. 4:**
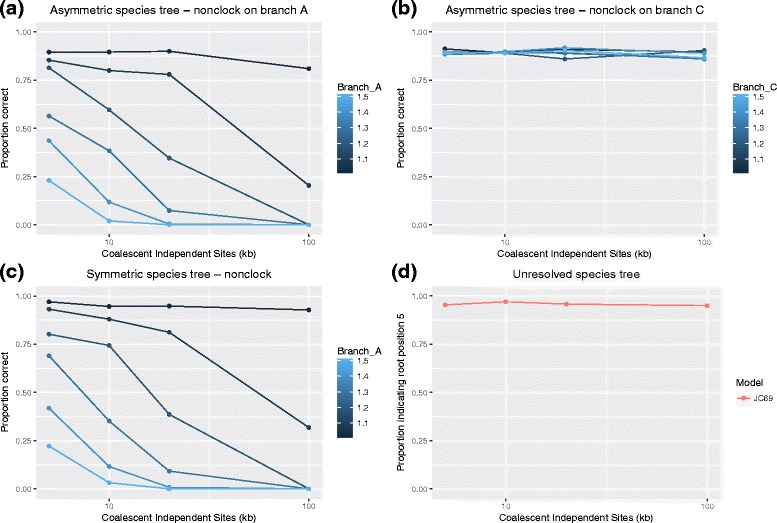
Accuracy of the rooting method when the molecular clock assumpon is violated. In each panel, the *x*-axis denotes the data size (number of coalescent independent sites in kb), and the *y*-axis shows the proportion of the data sets for which the correct rooting position is selected in a total of 500 simulations. **a** Asymmetric species tree with root position ① for which the branch leading to taxon *A* has been extended; **b** Asymmetric species tree with root position ① for which the branch leading to taxon *C* has been extended; **c** Symmetric species with root position ⑤ for which the branch leading to taxon *A* has been extended; **d** Proportion of times root position ⑤ is selected for the star phylogeny. All simulations used the JC69 model, since the first simulation study did not indicate systematic differences in performance based on varying the model

The power of the rooting method in the three simulation studies is shown in Figs. 3 and 4. In 500 simulations, the proportion of the data sets for which the correct rooting position is selected is summarized. The panels in the first column of Fig. 3 (panels (a) and (c)) represent the power for detecting the correct root positions for the simulation studies with coalescent independent sites. The panels in the second column (panels (b) and (d)) show the power for rooting phylogenetic trees in the second simulation set, where multi-locus DNA sequence data is simulated. Clearly, the simulation conditions that strictly follow the assumptions (free recombination and constant evolutionary rate) of the rooting method have very high power. When the assumption of free recombination is violated (e.g., for the multi-locus DNA sequence data in column 2), the tests have a slightly lower accuracy when the number of sites is small. Overall, it is safe to conclude that the new rooting method has high accuracy for rooting a four-leaf unrooted species tree. Notably, when the sample size is increased to about 10,000 bp, the accuracy is over 90% even for multi-locus DNA sequence data.

In our simulation studies, DNA sequence data are simulated under three different nucleotide substitution models: JC69, HKY85, and GTR+I+ *Γ* (labeled by black, red, and green in Fig. 3). Though the hypothesis tests for the rooting method are derived from the JC69 model, as described in the Methods section, the results of the simulation studies suggests that the rooting method can be applied to more general nucleotide substitution models. Over all of the conditions we tested, there was no systematic difference between the results for the JC69 model and for the other two models. Furthermore, the performance of our method in rooting the phylogenetic trees depends primarily on the sample size. More specifically, species trees with more coalescent independent sites or longer DNA sequences sampled can be rooted more accurately. As shown in Fig. 3, the solid lines denote the results for the species trees with “longer” branch lengths, and the dashed lines show the results for the species trees with “shorter” branch lengths. In general, the power for species trees with “longer” branch lengths is sightly higher, especially when the sample size is small (around 5000 bp). Thus, including more coalescent independent sites improves the accuracy of the test. Based on our simulations, around 10,000 bp for both long and short branch lengths are sufficient to ensure 95% accuracy when the data consist of coalescent independent sites, and 90% accuracy when multi-locus DNA data is used. Notably, the ability to identify the root of symmetric species trees does not depend on the sample size (Fig. 3 c and d), since the accuracy of identifying the root of symmetric species trees only relates to the significance levels that we selected for the hypothesis tests. The effects of sample size are not surprising, since the site pattern probabilities are estimated more accurately with more coalescent independent sites or longer DNA sequences, which is helpful in estimating the evolutionary relationships.

The results of the simulation studies for which the molecular clock assumption is violated are shown in Fig. 4 a - c. Figure 4 a and b show the power to detect the root for the asymmetric tree when the branch leading to taxon *A* or that leading to taxon *C* are extended, respectively, while Fig. 4 c shows the power when the symmetric tree is assumed and the branch leading to taxon *A* is extended. We can see that the power decreases as the amount of deviation from the molecular clock increases. It is also clear that the power decreases with increasing sample size, a result which at first seems counterintuitive. We discuss this further in the “Discussion” section.

Finally, Fig. 4 d gives the results of applying the rooting method to a star phylogeny (i.e., a phylogeny for which there is not a root). In this case, we might expect the method to identify branch ⑤ as the root, since the star tree will satisfy the two relationships that the symmetric tree induces and on which our hypothesis tests are based. Figure 4 d indicates that the procedure does indeed select the symmetric rooting about 95% of the time when a 5% significance level is used.

### Application to an eight-taxa North American rattlesnake data set

**Fig. 5:**
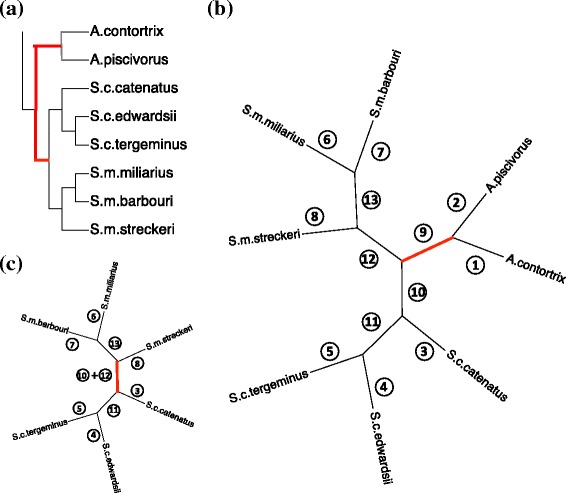
Application of the rooting method to the rattlesnake data set. **a** The 8-taxon species tree rooted by outgroups: *A. contortrix* and *A. piscivorus*. The inferred root position is labeled by a red line. **b** The unrooted 8-taxon species tree, with each branch labeled from ① to ⑬. The root position indicated by our method is labeled in red. **c** The unrooted 6-taxon species tree (with outgroups removed), with each branch labeled as in **b**. The rooting position indicated by our method is labeled in red

The simulation studies above show good accuracy and efficiency of the rooting method in identifying the root of a four-taxon species quartet. The next step is to examine the performance of our method for a larger empirical data set. We choose as a test case a data set of North American rattlesnakes that consists of samples from three subspecies of *Sistrurus catenatus* (*S. c. catenatus*, *S. c. edwardsii*, and *S. c. tergeminus*), three subspecies of *Sistrurus miliarius* (*S. m. miliarius*, *S. m. barbouri*, and *S. m. streckeri*), and two outgroups (*Agkistrodon contortrix* and *Agkistrodon piscivorus*). This is a multi-locus DNA data set with 19 genes and a total of 8466 base pairs. One individual is selected from each taxon to estimate the species tree and the root position. The estimated species tree is shown in Fig. 5 a, which is consistent with earlier analyses of Kubatko et al. [47] and Chifman and Kubatko [48]. With two known outgroups, *A. contortrix* and *A. piscivorus*, the putative root position is labeled in red lines in Fig. 5 a.

When the outgroups are unknown, the unrooted 8-taxon species tree estimated by SVDQuartets is shown in Fig. 5 b, with each branch labeled from ① to ⑬. A total of 70 species quartets within this species tree are examined to explore the root position based on our method, and the scores described in the Methods section are recorded for each branch (Table 3, “8-taxon”). We also removed the two outgroup species and tested our method with the remaining six taxa (Fig. 5 c), and record the scores in Table 3 (6-taxon). Note that the branches of the six-taxon species tree are given the same label as in the eight-taxon species tree (Fig. 5 b). Thus, branches ①, ②, and ⑨ no longer exist in Fig. 5 c.

**Table 3:** Rooting results for the North American rattlesnake data set

	①	②	③	④	⑤	⑥	⑦	⑧	⑨	⑩	⑪	⑫	⑬
8-taxon	9.67	9.67	0	0.25	0.25	0.25	0.25	0.33	31.00	7.42	1.75	7.42	1.75
6-taxon	NA	NA	0	0	0.25	0.25	0.25	0.33	NA	10.84 ^*a*^	1.42	NA ^*a*^	1.42

^*a*^For the 6-taxon species tree, the root position lies on the branch “⑩ + ⑫” (Fig. 5). The score for this branch is recorded under branch ⑩ in this table, and the score for branch ⑫ is thus labeled “NA”

From the scores of each branch (Table 3), it is easy to see that branch ⑨ should be selected as the root position for the eight-taxa species tree, which is consistent with previous analyses (Fig. 5 a). When the outgroups are removed from the analysis, our method can still accurately determine the root position on branch ⑩ and ⑫ (Table 3, “6-taxon”). Note that every single test of the 70 species quartets in the eight-taxon species tree correctly determined the root position, indicating an extremely high power for our method.

## Discussion

In this study, we develop a new method for rooting species-level phylogenies using site pattern probabilities. More specifically, our method roots the quartet species trees under the coalescent model, and then applies the results of rooted quartets to infer the root location in larger species trees. The accuracy of this method is examined by simulation studies and by application to an empirical North American rattlesnake data set. Notably, our method for rooting phylogenetic trees does not require specification of an outgroup, which makes it useful under very general conditions.

### Rooting phylogenetic trees under different nucleotide substitution models

For a given species tree, the probability distribution of all possible site patterns can be computed for different nucleotide substitution models (e.g., JC69, HKY85, GTR+I+ *Γ*, etc.). Specifically, for the simplest model, JC69, the identical base frequencies and the constant nucleotide substitution rate produce identical site pattern probabilities in many cases. For instance, given a four-taxon tree, there are only 15 unique site pattern probabilities under the JC69 model [45, 48]. That is to say, the site patterns that fall into the same category have identical probability, thus it is straightforward to use the mean of the site pattern probabilities within the same category to compute the test statistics we propose here.

More complex nucleotide substitution models, such as the HKY85 and the GTR+I+ *Γ* models, etc., can be specified by setting different rates for nucleotide changes. For example, HKY85 allows base frequencies to be unequal and considers one *transition* (substitutions between the two purines, *A* and *G*, or between the two pyrimidines, *C* and *T*) rate and one *transversion* (substitutions between a purine and a pyrimidine) rate, while the GTR model also allows unequal base frequencies, but defines a symmetric parameter-rich substitution matrix. Under these complex nucleotide substitution models, there will be a larger number of distinct site pattern probabilities and computing the probability of any site pattern probability will be more complex compared to the JC69 model. Indeed, the site pattern probability under the coalescent cannot be expressed as an analytic expression for the GTR+I+ *Γ* model, for example. However, the SVDQuartets method that is based on site pattern probabilities can still be applied to estimate a phylogenetic tree under models like HKY85 and GTR+I+ *Γ* [45, 48], and it is not difficult to show that our rooting method can be applied to phylogenetic data under these complex nucleotide substitution models, as well. Although there are no explicit formulas and the site pattern probabilities may not be identical within the 15 categories described here, the relationship between site pattern categories *yxxx* and *xyxx*, and between categories *xxyx* and *xxxy*, for example, will still hold. What changes is that the probabilities of patterns *ACAA* and *ATAA*, for example, may differ from one another under more complex models, even though they will still match *CAAA* and *TAAA*, respectively, when the clock holds. We have simulated sequence data under both the HKY85 and GTR+I+ *Γ* models in our simulation studies to verify that our method still applies under these complex nucleotide substitution models. Our results (Fig. 3) indicate that the method works equally well under the three different nucleotide substitution models, regardless of the equality of base frequencies and substitution rates between bases.

### Rooting phylogenetic trees using multi-locus DNA sequence data

Note that our rooting method assumes free recombination among the sites. In other words, it is designed for coalescent independent sites. However, previous simulation studies and real-data analyses also indicated good performance of SVDQuartets in analyzing multi-locus DNA sequence data. Also, SVDQuartets is suitable for the case of variable substitution rates across sites (i.e., substitution rates drawn from an arbitrary Gamma distribution) [49, 50]. The conclusion is similar for the rooting method presented here. As shown in Fig. 3, the method is highly accurate in identifying the root positions when varying substitution rates are drawn from an arbitrary Gamma distribution. Furthermore, the simulation studies that simulate multi-locus DNA sequence data also show good performance. This is quite reasonable, because under the coalescent model, the distribution of expected gene trees across loci for multi-locus DNA sequence data should be consistent with that obtained for independent sites, and thus the site pattern frequency distribution should be close to one another when each gene has a similar size. From Fig. 3, when there are more than 100 genes (10,000 bp in total), multi-locus DNA sequence data can be safely used to estimate rooted species tree directly from the site pattern probabilities.

### The molecular clock assumption

The method performs poorly when the molecular clock assumption is violated, as our test statistics are very sensitive to this assumption. Any deviation of the site pattern frequencies due to differing branch lengths is interpreted as evidence against a particular root location, and thus the tests become more likely to reject the correct root location as the sample size increases. Thus, we do not recommend that the method be applied when the assumption of a molecular clock is not reasonable. Though this limits the applicability of the method, we note that other rooting methods designed for gene trees (e.g., midpoint rooting and molecular clock rooting – see [35]) are also sensitive to this assumption. Because our method is the only method designed to accommodate the coalescent process, it contributes to the collection of methods available for rooting phylogenetic trees. It is an open question of whether test statistics that are not sensitive to the molecular clock assumption could be developed based on site pattern frequencies; we feel that this approach is promising.

### Control of familywise error rate

Controlling the familywise error rate appropriately when performing multiple hypotheses tests is a well-studied topic. In our method, we considered two hypothesis tests at the same time. To ensure a 95% confidence level, we choose to control the total Type I error at level 0.05. Using the Bonferroni correction [51], the significance level for each test is selected to be 0.025 in all of our simulation studies. Based on the hypothesis tests, when neither test can be rejected, we infer the symmetric species tree. Thus, the probability that a symmetric tree is inferred when the tree is indeed symmetric should exceed 95%, since the Bonferroni test is conservative when the test are not independent, as is the case here. Figure 3 c and d shows the results of correctly identifying the symmetric species tree. Obviously, with coalescent independent sites, the power of the tests is right about 95% on average, while for multi-locus DNA sequence data, the power of the tests is slightly lower than 95%, with larger variance. This can be explained by the violation of free recombination for multi-locus DNA sequence data. When each nucleotide is not independent from each other, it is reasonable for us to observe a larger variance and a slightly lower power. In general, even with multi-locus DNA sequence data, the power of our rooting method still exceeds 90%, indicating that this rooting method is an accurate and efficient way to locate the root position in a species tree.

Setting the significance level at 0.025 for both tests gives very good performance in all of our simulation studies. However, choosing different significance levels is also possible. In fact, we recommend that users select larger significant levels with a small sample size, and choose smaller significant levels with a huge data set. The relationship between margin of error and sample size is well-studied [52, 53]. Generally, larger sample sizes will lead to lower p-values [54, 55], thus requiring a smaller significance level. Additionally, the significance levels of the two hypothesis tests are not required to be identical. Once the sum of the two tests is smaller than 0.05, the overall error rate will be controlled at 5%. Thus, in general, differing significance levels can be picked for each test, depending on the relative importance for the application of interest.

## Conclusion

We have described a novel method for rooting phylogenetic species trees under the coalescent model. Our method works by rooting quartet trees, and then using these rooted quartet trees to infer the root location on a larger phylogeny. The method is shown to perform well for both simulated and empirical data when the molecular clock assumption holds, but is shown in simulation studies to be sensitive to this assumption. Because the method is based on the frequencies of observed site patterns, it is computationally efficient and thus provides a useful rooting method for species trees in the absence of outgroup information.

## Abbreviations

ILS: Incomplete lineage sorting
